# Fabrication of a novel porous nanostructure based on NiCuFe_2_O_4_@MCM-48, embedded with graphene oxide/poly (p-phenylenediamine) to construct an efficient supercapacitor

**DOI:** 10.1038/s41598-024-53241-7

**Published:** 2024-02-07

**Authors:** Zahra Sadat, Reza Eivazzadeh-Keihan, Vahid Daneshvari-Esfahlan, Samad Dalvand, Amir Kashtiaray, Ali Maleki

**Affiliations:** 1 Catalysts and Organic Synthesis Research Laboratory, Department of Chemistry, Iran University of Science and Technology, Tehran, 16846-13114 Iran; 2 Electrochemistry Research Laboratory, Department of Physical Chemistry, Faculty of Chemistry, University of Tabriz, Tabriz, 51666-16471 Iran; 3 Iranian Research & Development Center for Chemical Industries (IRDCI), Academic Center for Education, Culture and Research (ACECR), Karaj, Iran

**Keywords:** Chemistry, Materials science, Nanoscience and technology

## Abstract

In this study, a new nanocomposite was created by combining copper-doped nickel ferrite (NiCuFe_2_O_4_) nanoparticles with MCM-48 (Mobil Composition of Matter No. 48) on a graphene oxide (GO) substrate functionalized with poly(ρ-phenylenediamine) abbreviated as (PρPD). This nanocomposite was developed to investigate its potential for enhancing the function of a supercapacitor in energy storage. Following NiCuFe_2_O_4_@MCM-48 preparation, Hummer’s technique GO was applied. In-situ polymerization of NiCuFe_2_O_4_@MCM-48/GO nanoparticles with ρ-phenylenediamine (ρPD) in the presence of ammonium persulfate (APS) produced PρPD, a conductive polymer. Structural characterization of the nanocomposite includes FTIR, XRD, VSM, TGA-DTG, EDX, and FE-SEM. Results from BET indicate a pore size increase of up to 5 nm. Fast ion penetration and higher storage in capacitor material are explained by this. Additionally, the nanocomposite’s electrochemical performance was evaluated using GCD and CV tests. The NiCuFe_2_O_4_@MCM-48/GO/PρPD nanocomposite has a specific capacitance of 203.57 F g^−1^ (1 A g^−1^). Furthermore, cyclical stability is essential for energy storage applications. The nanocomposite retains 92.5% of its original capacitance after 3000 cycles, indicating outstanding electrochemical stability.

## Introduction

**Scheme 1:**
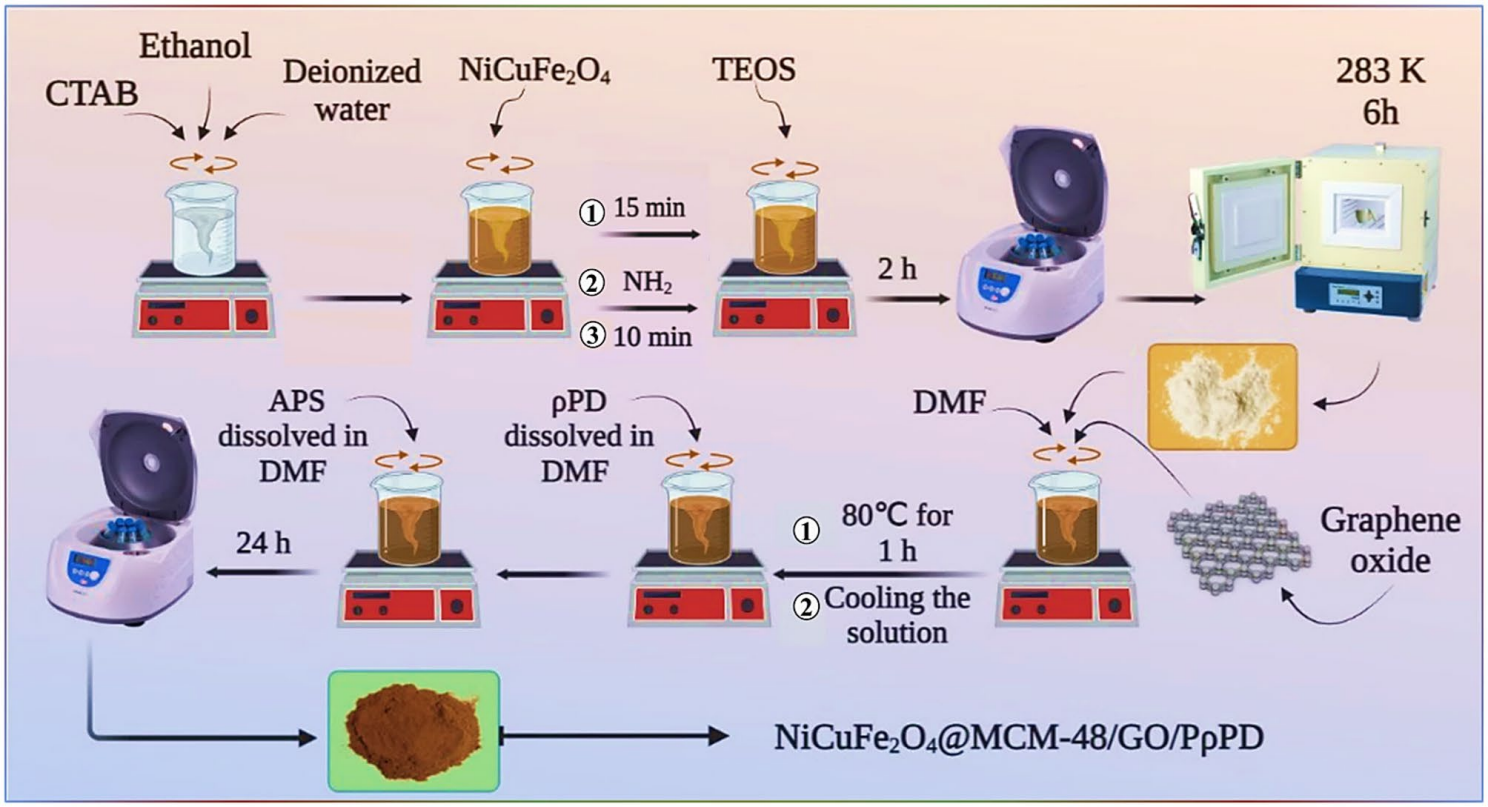
Preparation of NiCuFe_2_O_4_@MCM-48/GO/PρPD nanocomposite.

Excessive energy use and economic expansion have caused environmental contamination, endangering human health and natural systems. The need for alternative energy sources such as solar, wind, tidal, and nuclear power has arisen due to the conflict between fossil fuel burning and human energy needs. Efficient energy storage is crucial for successful energy management^1–3^. To choose the best energy storage option, consider variables such as durability, reliability, storage capacity, cost, and environmental effect. Supercapacitors, a potential energy storage technology, are popular for their compact size, lightweight, fast charge/discharge rates, high power density, and long cycle life^4^. In summary, supercapacitors store energy in two ways. The first technique, the electric double-layer capacitor (EDLC), involves ion adsorption on the electrode surface without chemical reactions. The second process, Faraday capacitance (sometimes called pseudocapacitance), involves fast redox reactions on the electrode’s surface^5^. Carbon electrode materials, such activated carbon, graphene oxide, and carbon nanotubes, are often utilized in supercapacitors, fuel cells, solar cells, and batteries owing to their porous structure and huge cross-sectional area. EDLC-type behavior is seen in these materials due to ion adsorption at the electrode contact^6^. Moreover, supercapacitor research has focused on graphene oxide (GO) due to its higher capacity of up to 189 F g^−1^, mechanical properties^7,8^, conductivity^9^, thermal properties^10^, biocompatibility^11–13^, low cost, easy processing^14^ and quasi-capacitive effect from oxygen-containing functional groups on baseplates^15,16^. However, for materials like metal oxides^9,17^ and conductive polymers (e.g., polyaniline^18^ and polypyrrole^19^), which exhibit quasi-capacitive behavior, charge storage is based on fast Faradic reactions^20^. Although the second mechanism stores more charge than the first, its cycle stability is minimized owing to lesser conductivity. To increase energy density and cycle stability without sacrificing power density, a hybrid technique combines carbon-based and quasi-capacitive materials^21^. Additionally, other materials may be used to enhance the characteristics of these compounds^22^. Porous compounds like MCM-48 are one kind^23^. The term “Mobil Composition of Matter” (MCM) was first used to refer to a group of mesoporous materials that were originally created by researchers at Mobil in 1992. MCM-41 and MCM-48, which stand for Mobil Composition of Matter No. 41 and Mobil Composition of Matter No. 48 respectively, are extensively researched mesoporous molecular sieves^24^. Porous carbon electrodes perform well due to their high electrical conductivity, charge accumulation surface area, large pore diameter, and pore connectivity enabling fast ionic movement and electrolyte wetting^25^. Additionally, their cheap cost, various forms, and chemical stability make them popular supercapacitor electrodes^26^. High specific capacitance in carbon electrodes demands increased surface area and porosity to improve composites’ surface-to-volume ratio and electrochemical reaction contact^27,28^. This study implanted NiCuFe_2_O_4_ nanoparticles with MCM-48 on a graphene oxide (GO) substrate functionalized with poly(p-phenylenediamine) (PρPD). This nanocomposite was researched for energy storage. Energy storage applications need cycling stability. The NiCuFe_2_O_4_@MCM-48/GO/PρPD nanocomposite maintains 92.5% of its original capacitance after 3000 cycles, demonstrating excellent electrochemical stability (Scheme 1).

## Experimental

### Materials and methods

The chemical materials, including reagents and solvents, used in this investigation were of high purity and purchased from international companies (Merck, Aldrich, and Fluka). Various analytical techniques were employed to study the structure of the nanocomposite. Fourier-transform infrared (FT-IR) spectroscopy (Shimadzu FT-8400 s model, Japan) was performed with a frequency range of 400–4000 cm^−1^ and using potassium bromide pellets for functional group identification. Field-Emission Scanning Electron Microscopy (FE-SEM) analysis (KYKY, EM8000 model) and Energy Dispersive X-Ray Analysis (EDX) analysis (BRUKER instrument) were used for morphology and elemental analysis, respectively. TGA/DTA was recorded using a BÄHR apparatus, X-ray diffraction analysis (XRD) was recorded with a BRUKER D8 ADVANCE device, and a N2 adsorption–desorption isotherm was recorded and analyzed using a MICROMERITICS ASAP 2020 instrument. Vibrating-sample magnetometer analysis (VSM) was carried out by Meghnatis Daghigh Kavir-MDKB-Kashan.

### Preparation of nanocomposite

#### Preparation of NiCuFe_2_O_4_

NiCuFe_2_O_4_ polycrystal is synthesized by a simultaneous precipitation method. In the first place, a 0.5 M solution was made separately from each of the salts, namely nickel nitrate, copper nitrate, and iron nitrate. Afterward, all three solutions were poured into the flask for stirring until all the salts were completely dissolved. In the next step, 0.2 M NaOH solution was added to the flask at a temperature of 60 °C, and then the temperature was raised to 100 °C and stirred for 6 h. Subsequently, the nanoparticles that were initially deposited were subjected to filtration and centrifugation, followed by drying in an oven at a temperature of 60 °C for a duration of 48 h. Finally, the obtained nanoparticles were ground in an agate mortar and calcined for 5 h at a temperature of 600 °C inside the furnace.

#### Preparation of NiCuFe_2_O_4_@MCM-48

For the in-situ synthesis of NiCuFe_2_O_4_@MCM-48, 2.4 g (6.6 mmol) of *n*-hexadecyltrimethylammonium bromide (CTAB) as a template was dissolved in 50 g of deionized water and 28 g (0.87 mol) of industrial ethanol. Then, after adding 0.5 g of NiCuFe_2_O_4_ and stirring the mixture for 15 min, 3.4 g (0.2 mol) of ammonia was added to the surfactant solution. After stirring the solution for 10 min, 3.4 g of TEOS were added and the mixture was stirred for an additional 2 h at ambient temperature. The solid material was then obtained by filtration, washed with distilled water through centrifugation, and dried at ambient temperature. To remove the template, the material was placed in a furnace for 6 h at a temperature of 823 K (equivalent to 550 °C).

#### Preparation of GO

The preparation of graphene oxide involved a modified Hummer method, as described in previous studies^12^. To begin, 1 g of graphite was combined with 23 ml of 98% sulfuric acid in a 1000 ml beaker. The mixture was stirred for a few minutes before adding 0.5 g of sodium nitrate (NaNO_3_). The stirring continued for 25 min at a temperature of 66 ℃. To ensure the complete dissolution of components, the beaker was subjected to an ultrasonic bath at room temperature for 30 min. Following that, 3 g of potassium permanganate were gradually added over a period of 1 h with continuous stirring, while the beaker was maintained in an ultrasonic ice bath. This process continued until a dark green, sludge-like substance was formed. To complete the reaction, the mixture was stirred for an additional 30 min in the ultrasonic bath at room temperature. Next, 50 ml of distilled water were added, and the mixture was stirred for 30 min at a temperature of 98 °C. To induce a significant amount of foam formation, 700 ml of distilled water and 12 ml of hydrogen peroxide were added simultaneously. Afterwards, the pH of the mixture was adjusted by adding a 2% solution of hydrochloric acid (2 ml of HCl per 100 ml of distilled water). The mixture was left to settle for 1 day, following which the water in the beaker was replaced and the elution process was repeated three times. Ultimately, the precipitate was dried in an oven at a temperature of 60 ℃ for a duration of 24 h.

#### Preparation of NiCuFe_2_O_4_@MCM-48/GO

To load NiCuFe_2_O_4_@MCM-48 onto GO sheets, in a 250 ml flask, 0.07 g of NiCuFe_2_O_4_@MCM-48, along with 0.4 g of GO, was stirred in 100 ml of DMF at 80 °C for 1 h.

#### Preparation of NiCuFe_2_O_4_@MCM-48/GO/PρPD

During the final stage, the temperature was lowered before introducing a solution consisting of 0.4 g of ρPD dissolved in 20 ml of DMF into the previously prepared mixture. While maintaining the flask in an ice bath, a solution of 2 g of APS in 40 ml of DMF was gradually added dropwise. The mixture was then stirred for 24 h at the same temperature, and the resulting product was obtained through a series of washing steps using ethanol and distilled water (repeated twice), followed by centrifugation and drying at room temperature.

## Results and discussion

### Characterization

#### XRD pattern

**Figure 1:**
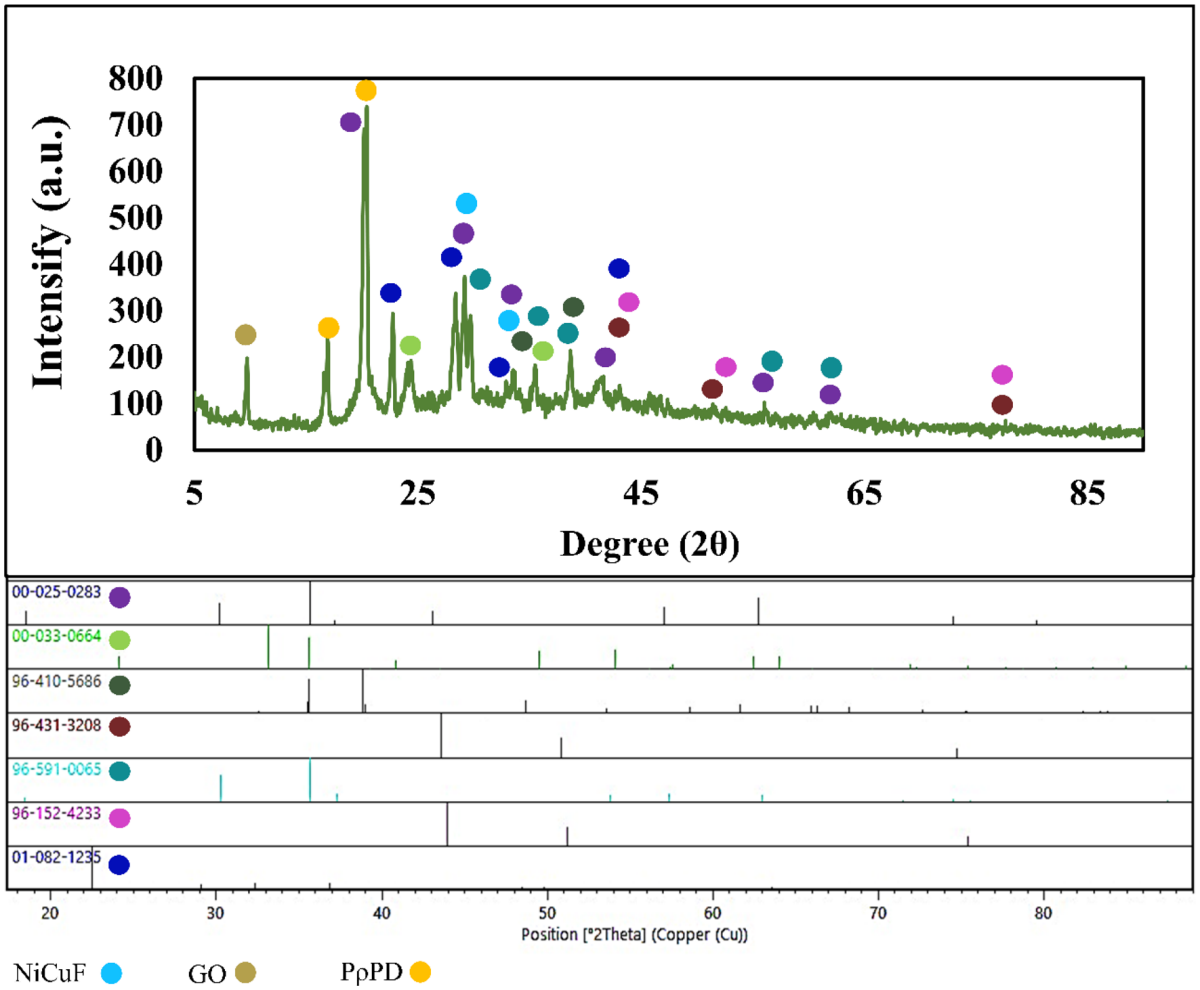
XRD pattern of NiCuFe_2_O_4_@MCM-48/GO/PρPD.

The XRD pattern of NiCuFe_2_O_4_@MCM-48/GO/PρPD is shown in Fig. 1. The peaks at 2θ are characteristic of the ferrite material, with the highest peak at 35.676° (311), confirming the formation of the cubic spinel structure^29^. The cubic phase of spinel ferrites (NiCuFe_2_O_4_) is characterized by the presence of peaks indexed as 30.16° (220), 35.64° (311), 43.03° (400), 57.05° (511), 62.77° (440), and 74.54° (533) according to the JCPDS Card No: 00-025-0283^30–32^. The identified peaks in 2θ correspond to NiFeO (35.676° (131), 30.287° (022), 37.320° (222), 57.361° (151), 62.997° (044)), Cu (43.621° (111), 50.810° (020), 74.706° (022)), CuO (38.854° (111), 35.571° (− 111), 48.708° (-202), 35.543° (002)), NiCu (43.96 (111), 51.22 (020), 75.37 (022)), NiCuF (35° (311), 31° (220)), and Fe_2_O_3_ (24.13° (012), 33.153° (104), 35.612° (110), 49.480° (024), 54.091° (116)). The identified peaks match with the ICDD (International Diffraction Data Center) cards (96-591-0065), (96-431-3208), (96-410-5686), (96-152-4233)^33^, and (00-033-0664), respectively. The peaks at 2θ = 22.51°, 29.09°, 32.34°, 36.83°, and 43.6° are attributed to the tetragonal SiO_2_ phase of MCM-48 (JCPDF: 01-082-1235)^34^. Two distinct XRD peaks at approximately 2θ = 17° and 21° were observed for PρPD^35,36^, while the main and well-defined diffraction peak of GO was identified at 2θ = 10° (001)^37^.

#### Fourier-transform infrared spectra

**Figure 2:**
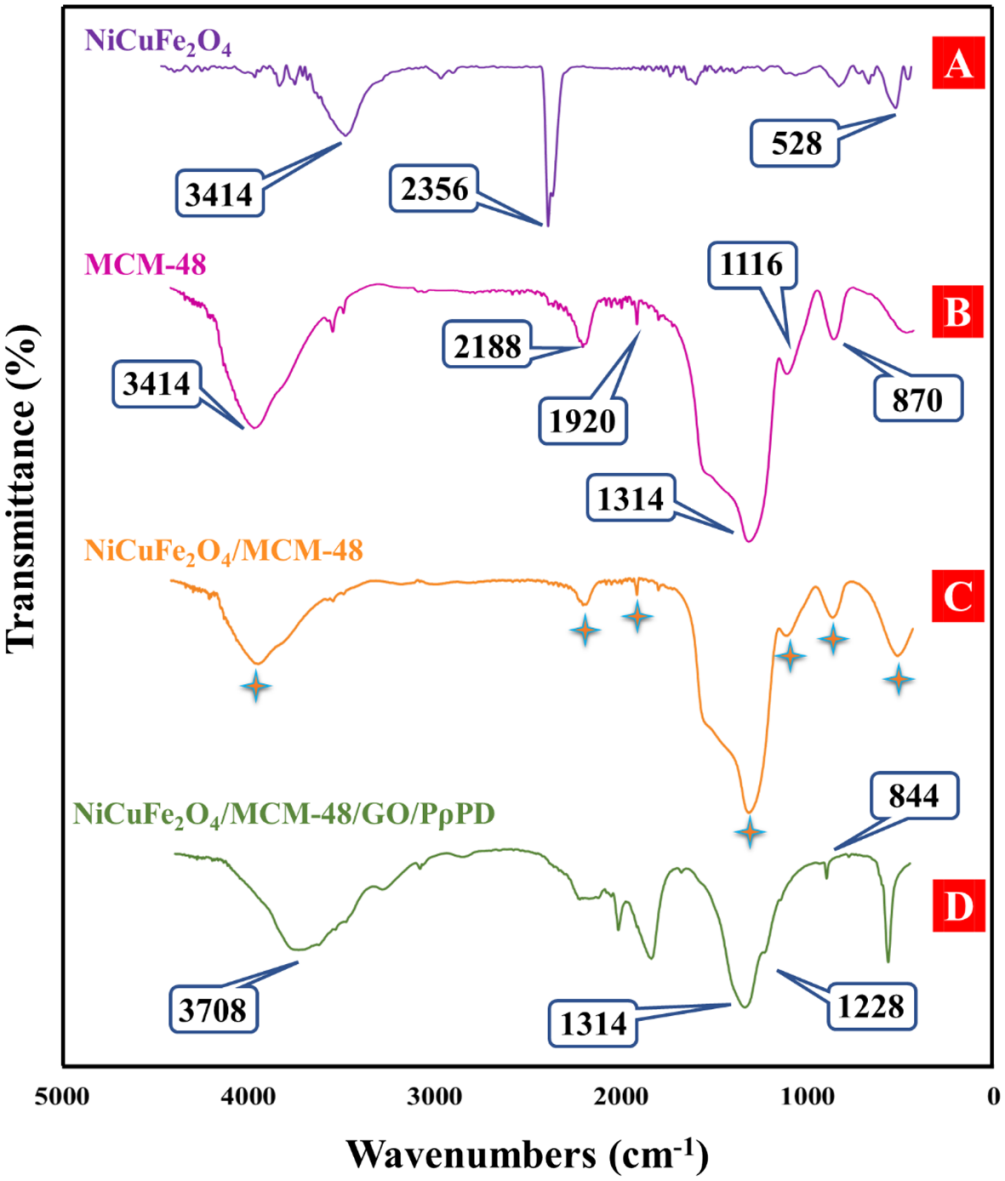
Fourier-transform infrared (FTIR) spectra of samples.

Fourier transform infrared (FTIR) spectroscopy was performed on NiCuFe_2_O_4_ (Fig. 2A), MCM-48 (Fig. 2B), NiCuFe_2_O_4_@MCM-48 (Fig. 2C), and NiCuFe_2_O_4_@MCM-48/GO/PρPD (Fig. 2D) in the range of 4400–440 cm^−1^. The sharp peaks associated with MCM-48 were consistent with previous reports. A wide absorption band that appears within the range of 3400–3900 cm^−1^ is typically linked to the presence of Si (OH), Si (OH)_2_, or Si (OH)_3_ groups on the surface of the pores. The peak located at 2188 cm^−1^ corresponds to the stretching vibration of Si–OH, while the band situated at 1314 cm^−1^ is associated with an asymmetric stretching vibration of Si–O–Si. The band located around 870 cm^−1^ is attributed to the symmetric stretching vibration, and the peak situated at 460 cm^−1^ corresponds to the bending vibration of Si–O–Si^38–41^. The NiCuFe_2_O_4_ spectrum exhibited a band centered around 475 cm^−1^, which can be attributed to the stretching vibration of a metal–metal (Ni–Cu) band^42,43^. In the case of spinel ferrites, FTIR bands resulting from ion vibrations within the crystal lattice usually appear in the range of 1000–100 cm^−1^. The band at a higher frequency (555–600 cm^−1^) and the band at a lower frequency (v2) (480–495 cm^−1^) are attributed to the tetrahedral and octahedral complexes of spinel ferrites, respectively. The obtained spectrum indicated a band at approximately 528 cm^−1^. These intense absorption bands are characteristic of inverted spinel ferrites^44^. Bands with wavenumbers below 400 cm^−1^ were not clearly observed due to limitations of our FTIR instrument. The band situated at 3414 cm^−1^ could be related to the stretching vibration of the –OH group, potentially present as a result of adsorbed water molecules. Additional bands were identified at 2918, 2849, 2356, and 1384 cm^−1^, which may correspond to C–H stretching modes linked to surfactant molecules attached to the NiCu alloy nanoparticles^45,46^. The absorption band located at 600 cm^−1^ may be assigned to the presence of copper ferrite CuFe_2_O_4_ within the sample^47^. The spectrum of NiCuFe_2_O_4_@MCM-48 displayed all the peaks that were present in the spectra of NiCuFe_2_O_4_ and MCM-48. In the FTIR spectrum of NiCuFe_2_O_4_@MCM-48/GO/PρPD, the broad peak observed in the 3400–3750 cm^−1^ region is assigned to the stretching vibration mode of hydroxyl groups in GO and the stretching mode of the N–H of the secondary amine group in the polymer chain of PρPD. Two peaks are also visible at approximately 1314 and 1228 cm^−1^, which correspond to the stretching vibrations of the quinone imine units (–C–N– and C–N))^10,20,48–51^.

#### Field-emission scanning electron microscopy

**Figure 3:**
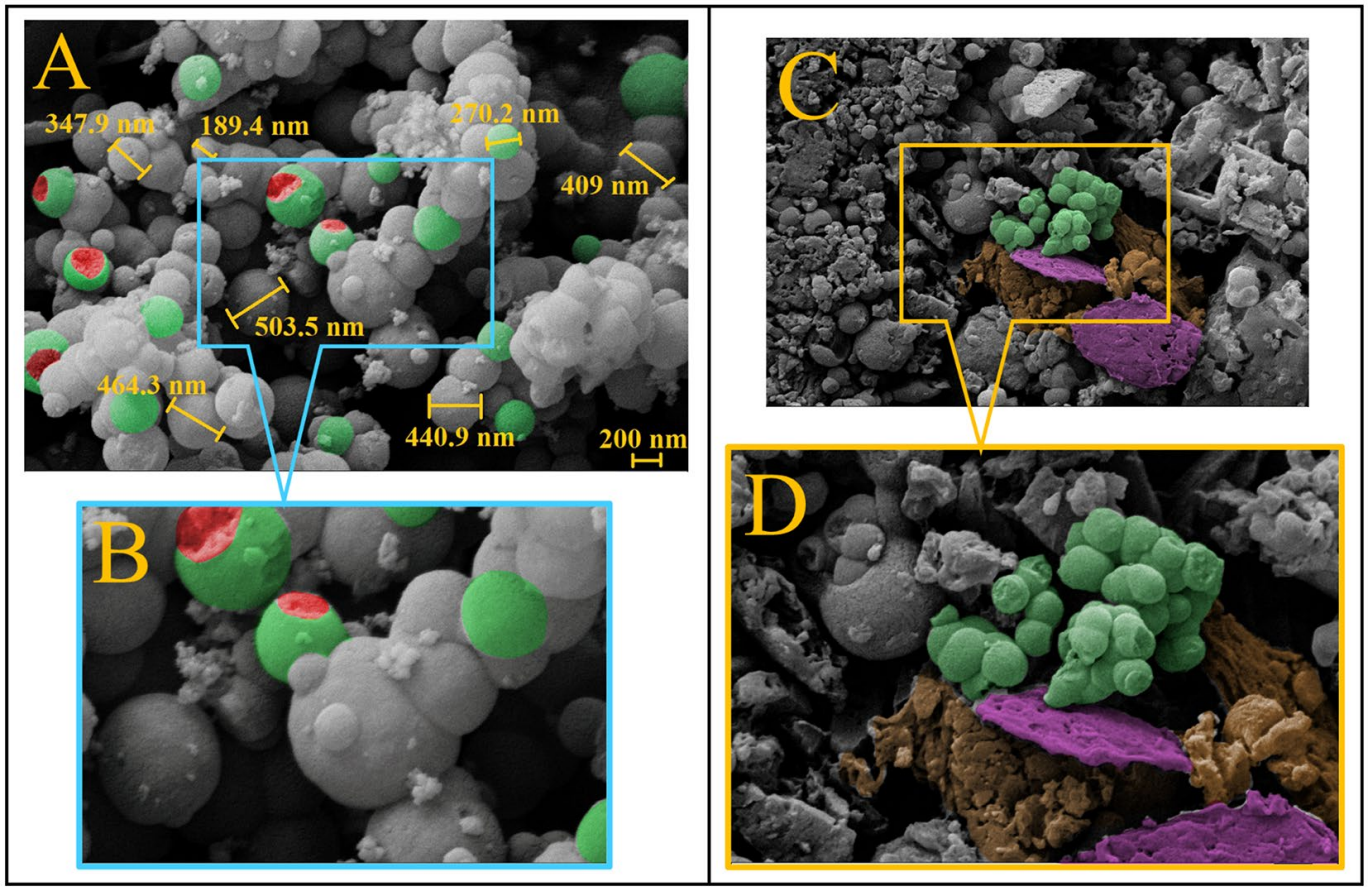
FE-SEM image of (**A**) NiCuFe_2_O_4_@MCM-48, (**B**) zoomed image of (**A**), (**C**) NiCuFe_2_O_4_@MCM-48/GO/PρPD and (**D**) zoomed image of (**C**).

Figure 3A depicts the morphology and structure of MCM-48 quasi-spherical particles, with a dominant particle size of approximately 100–500 nm^52^. The NiCuFe_2_O_4_ nanoparticles are well-covered by the MCM-48 silicate structure, although some parts of the image show the nanoparticles uncovered or inside cracked MCM-48 spheres. Further, Fig. 3B provides a zoomed-in view of Fig. 3A. The morphology of the final product can be observed in Fig. 3C, which shows NiCuFe_2_O_4_-covered nanoparticles with MCM-48, composite GO sheets, and PρPD polymer strands forming a matrix. Furthermore, Fig. 3D is a zoomed-in image of Fig. 3C.

#### Energy-dispersive X-ray spectroscopy

**Figure 4:**
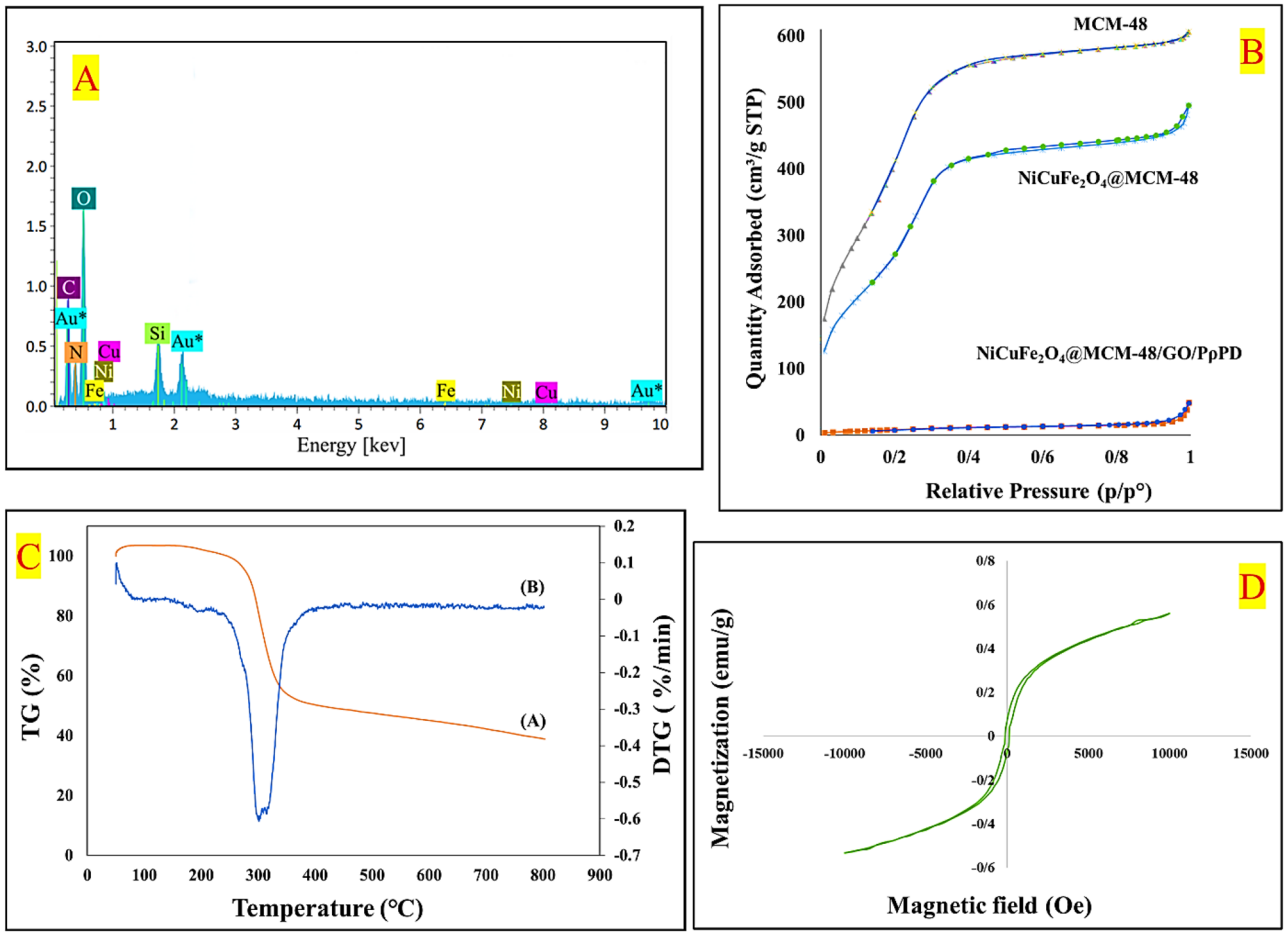
EDX spectrum of NiCuFe_2_O_4_@MCM-48/GO/PρPD (**A**), N_2_ adsorption–desorption isotherm of MCM-48, NiCuFe_2_O_4_@MCM-48 and NiCuFe_2_O_4_@MCM-48/GO/PρPD (**B**), The TGA, DTG curve (**C (A), (B)**) and VSM (**D**) of NiCuFe_2_O_4_@MCM-48/GO/PρPD.

Based on the results obtained from the EDX spectrum of this nanocomposite in Fig. 4A, the existence of NiCuFe_2_O_4_ nanoparticles covered by MCM-48 was confirmed by observing peaks for Nickel, Copper, Iron, oxygen, and Silicon. Besides, the presence of GO sheets and PρPD polymer was confirmed by observing three peaks of carbon, nitrogen, and a sharpened peak of oxygen.

#### BET analysis

Figure 4B shows the Nitrogen adsorption–desorption isotherms for MCM-48, NiCuFe_2_O_4_@MCM-48, and NiCuFe_2_O_4_@MCM-48/GO/PρPD. The isotherms display a type IV adsorption–desorption behavior with a clearly distinguishable hysteresis loop, which is characteristic of mesoporous materials^53^. The information in Table 1 shows that adding GO sheets and PρPD polymer to the NiCuFe_2_O_4_@MCM-48 composite material that was made on-site led to a gradual decrease in both surface area and pore volume compared to pure MCM-48. The presence of organic groups led to pore blockages, reducing the surface area of the mesoporous product^52^. Meanwhile, the pore size gradually increased due to the continuous decrease in micropores, and rich mesoporous structures formed. Over time, the pore size increased gradually as a result of a continuous decrease in micropores and the development of rich mesoporous structures. It is worth noting that high surface area does not necessarily translate to high capacitance values, and this holds true for all pore types^54^. The microporous structure provides channels for ion transfer, lowers ion-transport resistance, and, in turn, facilitates charge storage^55,56^.

**Table 1:** Textural properties of the samples.

Material	BET surface area (m^2^/g)	Pore volume (cm^3^/g)	Pore size (nm)
MCM-48	1583.7447	0.921556	2.32754
NiCuFe_2_O_4_@MCM-48	1022.6427	0.718166	2.80906
NiCuFe_2_O_4_@MCM-48/GO/PρPD	31.2834	0.039290	5.02375

#### Thermogravimetric analysis (TGA)

Thermogravimetric analysis (TGA) was performed under an argon atmosphere over the temperature range of 50–900 °C, as depicted in Fig. 4C(A). The weight loss observed during the heating process can be divided into three stages. In the first stage, there was a minor weight loss of around 3% at temperatures below 100 °C, which is likely due to the physical adsorption of water on the external surface or the removal of water from the mesoporous channels^57^. The second stage exhibited a sharp drop in weight, related to the removal of organic components at a temperature below 400 °C (with a decomposition rate of approximately 50% by weight)^58^, which was also confirmed by DTG curve Fig. 4C(B). The reduction in mass between 200 and 300 °C can be attributed to the thermal decomposition of oxygen-containing functional groups present in GO, such as carboxyl, epoxide, and hydroxyl groups^12^. Furthermore, the thermal degradation of PρPD polymer chains likely begins at a temperature range of 250 to 340 °C^59^. The final stage is observed in the temperature range of 400–600 °C and corresponds to the elimination of carbonaceous residues^58^.

#### Vibrating‑sample magnetometer (VSM)

A vibrating-sample magnetometer (VSM), shown in Fig. 4D, was used to check the magnetic properties of the NiCuFe_2_O_4_@MCM-48/GO/PρPD that was synthesized. The magnetization of the synthesized composite was measured at approximately 1.56 emu g^−1^. Compared to other research on NiCuFe2O4 nanoparticles, the composite has a relatively low magnetic saturation^60^. This may be because the nanoparticles are confined within the MCM-48 porous material and are surrounded by polymer and GO sheets.

#### Electrochemical measurements

The electrochemical experiments were carried out using a Autolab PGST200 electrochemical analyzer in a routine three-electrode configuration, with an Ag/AgCl reference electrode and a Platinum wire counter electrode. The working electrode was prepared by mixing the electroactive material (NiCuFe_2_O_4_@MCM-48/GO/PρPD), acetylene black, and polytetrafluoroethylene (PTFE) (80:10:10 wt%) in NMP under ultrasonic conditions. The resulting ink was then sprayed onto a stainless-steel mesh, followed by thermal treatment at 120°C. The capacitive behavior of the synthesized materials was studied in 1.0 M H_2_SO_4_ solutions. In the three-electrode system, cyclic voltammetry tests were carried out over a potential range of − 0.2 V to 0.8 V at various scan rates from 1 to 10 mV s^−1^. Additionally, galvanostatic charge–discharge measurements were conducted at different current densities (1–5 A g^−1^) with a potential window of − 0.2 to 0.8 V.

### Electrochemical application of the NiCuFe_2_O_4_@MCM-48/GO/PρPD

**Figure 5:**
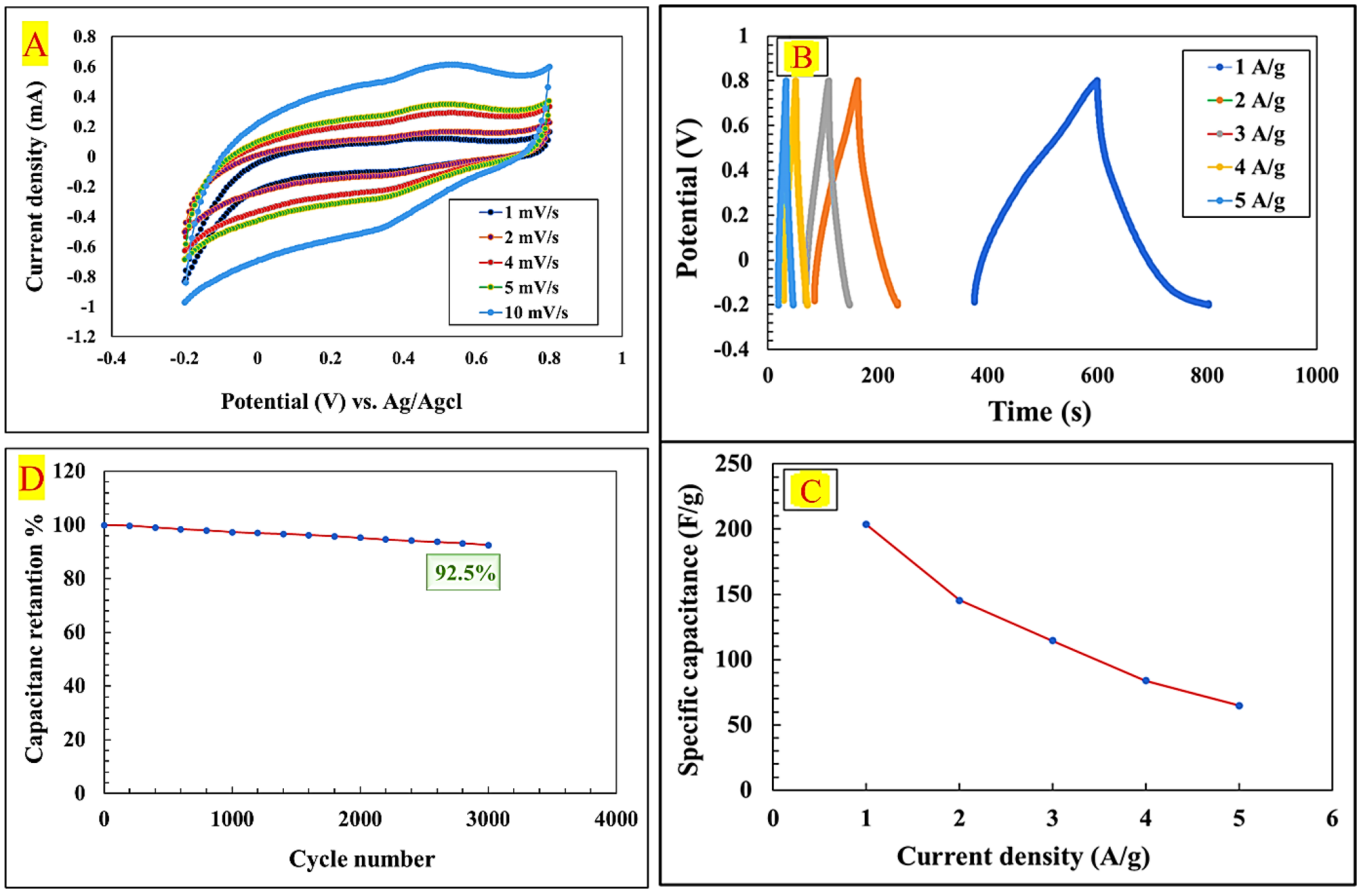
CV curves of NiCuFe_2_O_4_@MCM-48/GO/PρPD at various scan rates in 1.0 M H_2_SO_4_ electrolyte solutions (**A**), GCD curves (second cycle) of NiCuFe_2_O_4_@MCM-48/GO/PρPD at different current densities in 1.0 M H_2_SO4 electrolyte solutions (**B**); variation of the specific capacitance values versus the current density (**C**) and cycling stabilities of the NiCuFe_2_O_4_@MCM-48/GO/PρPD over 3000 GCD 1.0 M H_2_SO_4_ electrolyte solutions (**D**).

The electrochemical behavior of the NiCuFe_2_O_4_@MCM-48/GO/PρPD electrode was analyzed by performing cyclic voltammetry (CV) measurements in 1.0 M H_2_SO_4_ electrolyte within the potential window of − 0.2 to 0.8 V (vs. AG/AgCl) at varying scan rates ranging from 1.0 to 10 (mV s^−1^). As illustrated in Fig. 5A, the resulting CV curves display anodic and cathodic peaks in a quasi-rectangular shape, suggesting the existence of both electrical double-layer capacitors (EDLC) and pseudocapacitive behavior in the synthesized material. The distinctive redox peaks observed in the cyclic voltammogram can be attributed to the Faradaic redox reactions taking place at the surface of the electrode material. As the scan rate increases, it becomes apparent that the position of the anodic and cathodic peaks shifts towards more positive and negative potentials, respectively. This polarization phenomenon implies that the transfer of electrolyte ions onto the surface of the material is restricted at higher scan rates. The shape of the cyclic voltammogram remains relatively unchanged at different scan rates within the measured potential window, indicating the excellent rate capability of the electrode materials^61,62^.

To assess the storage capacitance of the nanocomposite, galvanostatic charge–discharge (GCD) test was conducted. The resulting GCD curves (second cycle), shown in Fig. 5B, were obtained at different current densities (1–5 A g^−1^) in 1.0 M H_2_SO_4_ electrolyte within the potential range of − 0.2 to 0.8 V. The GCD cycles exhibited nonlinear characteristics, suggesting that the capacitive behavior of the electrode was due to a combination of electrical double layer and pseudocapacitive processes^63^. As expected, the nonlinear shape of the charge/discharge curves is due to the quasi-capacitive behavior of metal oxides caused by the electrochemical reaction of adsorption–desorption or redox at the electrode–electrolyte interface. As is clear from the curves, discharge/charge times are almost parallel, indicative of good coulombic efficiency and electrochemical reversibility of NiCuFe_2_O_4_@MCM-48/GO/PρPD nanocomposite. It is also worth noting that the IR drop remains unchanged as the current density increases, indicating the exceptional capacitor performance and the reversible nature of the electrode materials, the results being in perfect agreement with the CV curves.

Specific capacitance values were calculated using Eq. (1) for various current densities.

1 $${c}_{s}=\frac{I\Delta t}{m\Delta v}.$$

The following symbols are used: C_s_ (F g^−1^) for specific capacitance, I (A) for discharging current, $\Delta t (s)$ for discharge time, m (g) for the mass of the electrode material, and $\Delta v$ (V) for potential difference. Figure 5C illustrates the specific capacitance of NiCuFe_2_O_4_@MCM-48/GO/PρPD electrode materials at various current densities. As the current density increases, the specific capacitance decreases due to restricted ion diffusion into the active sites. Specifically, the specific capacitance declined from 203.57 F g^−1^ at 1 A g^−1^ to 64.85 F g^−1^ at 5 A g^−1^.

The longevity of an electrode material is crucial for energy storage applications. To assess the cycling stability of the synthesized electrode materials in 1.0 M H_2_SO_4_, cyclic tests were performed at a current density of 3 A g^−1^, as illustrated in Fig. 5D. The results demonstrate that the electrode material’s capacitance retention after 3000 cycles is 92.5% of its original capacitance, indicating the exceptional electrochemical stability of the NiCuFe_2_O_4_@MCM-48/GO/PρPD electrode materials.

Table 2 presents a comparison between the capacitor ability of the NiCuFe_2_O_4_@MCM-48/GO/PρPD nanocomposite and other materials reported in recent research. The results reveal the high specific capacitance of the nanocomposite compared to each of its components in other compounds, along with its exceptional ability to maintain the initial capacity for reuse. Therefore, this makes it a potential candidate for use in supercapacitor applications.

**Table 2:** Comparison of capacitor ability of materials with NiCuFe_2_O_4_@MCM-48/GO/PρPD nanocomposite.

Material	Specific capacitance (F g^−1^)	Electrolyte	Maintain capacitance (%)	Ref.
NiCuFe_2_O_4_@MCM-48/GO/PρPD	203.57	1 M H_2_SO_4_	92.5	This work
Mesoporous fullerene C_70-150_	172	6.0 mol l^−1^ KOH	83.1	^64^
Hollow mesoporous carbon spheres-P	168	6.0 mol l^−1^ KOH	75.5	^65^
Nitrogen-containing activated porous carbon nanostructure (PNAC) derived from poly (para-phenylenediamine)	162	1 M H_2_SO_4_	–	^66^
GO	60	1 M H_2_SO_4_	–	^67^
NiCuF	16.9	1 M KOH	–	^44^

## Conclusion

To summarize, a composite material consisting of NiCuFe_2_O_4_ coated with MCM-48, GO nanosheets, and PρPD (produced by an in-situ technique) has been created and used as an electrode substance in supercapacitor applications. Using NiCuFe_2_O_4_ as a transition metal oxide along with MCM-48 porous carbon material improves the electrochemical efficiency of composite electrodes by adding redox centers that make it easier for charge to be stored through redox reactions. GO demonstrates a significant capacitance in capacitive applications because to the supplementary quasi-capacitive impact of oxygen-containing functional groups bonded to its base plates. Moreover, PρPD is an electrically conductive polymer capable of undergoing redox reactions with an electrolyte. These qualities provide them advantageous options for electrode components in supercapacitors. Analytical methods including FT-IR spectroscopy, XRD, VSM, TGA-DTG, EDX, and FE-SEM were used to assess the physical and chemical characteristics of the NiCuFe_2_O_4_@MCM-48/GO/PρPD nanocomposite. According to the findings from BET analysis, the produced nanocomposite has a maximum pore size of 5 nm. This supports the need for establishing channels that enable rapid ion transport with little resistance, hence enhancing the capacity for charge storage. The nanocomposite materials were analyzed for their electrical supercapacitive performance using cyclic voltammetry (CV), galvanostatic charge/discharge (GCD) experiments, and a cyclic stability investigation of the electrode. The composite exhibits a specific capacitance of 203.57 F g^−1^ (at 1 A g^−1^) and retains 92.5% of its original capacitance after 3000 cycles, indicating its suitability for supercapacitor applications.

## Data Availability

The whole of the data produced or examined during this investigation is included within this published publication.
